# Bloodstream infections in neutropenic and non-neutropenic patients with haematological malignancies: epidemiological trends and clinical outcomes in Queensland, Australia over the last 20 years

**DOI:** 10.1007/s10238-023-01206-x

**Published:** 2023-10-10

**Authors:** Anna Maria Peri, Felicity Edwards, Andrea Henden, Patrick N. A. Harris, Mark D. Chatfield, David L. Paterson, Kevin B. Laupland

**Affiliations:** 1 University of Queensland Centre for Clinical Research (UQCCR), Building 71/918 RBWH Herston, Brisbane City, QLD 4029 Australia; 2 Queensland University of Technology (QUT), Brisbane, QLD Australia; 3 Department of Haematology and Bone Marrow Transplantation, Royal Brisbane and Women’s Hospital, Brisbane, QLD Australia; 4 School of Medicine, University of Queensland, Brisbane, QLD Australia; 5 Central Microbiology, Pathology Queensland, Royal Brisbane and Women’s Hospital, Herston, Brisbane City, QLD 4029 Australia; 6 ADVANCE-ID, Saw Swee Hock School of Public Health, National University of Singapore, Singapore, Singapore; 7 Intensive Care Unit, Royal Brisbane and Women’s Hospital, Brisbane, QLD Australia

**Keywords:** Bloodstream infection, Haematological neoplasms, Neutropenia, Antimicrobial resistance, Epidemiology

## Abstract

Knowledge of the epidemiology of bloodstream infection (BSI) in haematology patients is essential to guide patient management. We investigated the epidemiology of BSI in patients with haematological malignancies in Queensland over the last 20 years (2000–2019), including all episodes diagnosed by the state-wide microbiology service. We identified 7749 BSI in 5159 patients, 58% associated with neutropenia. Gram-negatives were the main causative pathogens (58.3%), more frequent in neutropenic than non-neutropenic patients (3308/5309, 62.3% vs 1932/3678, 52.5%, *p* < 0.001). Amongst 8987 isolates the most common were *E.* *coli* (15.4%) and *Pseudomonas* spp. (14.2%). *Pseudomonas* spp. (16.6% vs 10.7%, *p* < 0.001), *Klebsiella* spp. (11.6% vs 6.8%, *p* < 0.001), viridans-group streptococci (4.4% vs 1.2%, *p* < 0.001) and *E.* *faecium* (2.4% vs 0.9%, *p* < 0.001) were more common in neutropenic than non-neutropenic patients, while *S.* *aureus* was less common (5.9% vs 15.6%, *p* < 0.001). Several antimicrobial resistance rates increased over time and had higher prevalence in neutropenic than non-neutropenic patients, including ciprofloxacin-resistant *E.* *coli* (94/758, 12.4% vs 42/506, 8.3%, *p* = 0.021), trimethoprim-sulfamethoxazole-resistant *E.* *coli* (366/764, 47.9% vs 191/517, 36.9%, *p* < 0.001), penicillin-resistant streptococci (51/236, 21.6% vs 28/260, 10.8%, *p* < 0.001) and vancomycin-resistant enterococci (46/250, 18.4% vs 9/144, 6.3%, *p* < 0.001). Carbapenem-resistant *Pseudomonas* spp. (OR 7.32, 95%CI 2.78–19.32) and fungi, including yeasts and moulds (OR 3.33, 95%CI 2.02–5.48) were associated to the highest odds of 30-day case-fatality at a multivariable logistic regression analysis. Neutropenia was associated with survival (OR 0.66, 95%CI 0.55–0.78). Differences were observed in the BSI epidemiology according to neutropenic status, with an overall increase of resistance over time associated to adverse outcome.

**Supplementary Information:**

The online version contains supplementary material available at 10.1007/s10238-023-01206-x.

## Introduction

Patients with haematological malignancies are at high risk of bloodstream infections (BSI) which represent one of the most important complications of anticancer therapy, associated with mortality rates up to 40% [1–3]. Neutropenia is a considerable risk factor for BSI that can sometimes present as isolated fever due to the impaired immune response. BSI occur in 10%–25% of neutropenic patients with fever and the incidence can be even higher in bone marrow transplant recipients (13–60%) [1].

The epidemiology of BSI among patients with haematological malignancies during neutropenia has been characterized by substantial changes over time. Gram-negatives were the most frequent isolates during the 1970s and early 1980s, while Gram-positives became the most prevalent in the late 1980s and 1990s due to the increased use of intravascular catheters, fluoroquinolone prophylaxis and anti-Gram-negative empirical treatment protocols [4]. However, during the last 20 years, Gram-negatives are re-emerging as the most prevalent pathogens in this population [5, 6]. Consistently, an increase in antimicrobial resistance has been reported in international and national cohorts of neutropenic patients [2, 5–7] leading to concerns over the best management of antimicrobial prophylaxis in this setting [8]. Differently, the epidemiology of BSI in non-neutropenic haematology patients has been characterized by less changes over time [2, 6].

Knowledge of the epidemiology of BSI in patients with haematological neoplasms is important to improve the understanding as to how this significant complication of therapy may arise, and to inform clinical decision making that may in turn contribute to improve patients’ management, including a better definition of protocols for antimicrobial prophylaxis and empirical treatment [9]. However, there is a major limitation in the current studies that have been published in the past several decades which are often limited to a single-centre design with small numbers of patients [3, 5, 7] and to surveillance periods shorter than 10 years [2, 3, 6, 7]. Many of these studies have focused on neutropenic patients [5, 7] and there is a scarcity of recent studies comparing BSI episodes in neutropenic and non-neutropenic patients [2, 6, 10]. Furthermore, many of these studies have been conducted in areas with high prevalence of antimicrobial resistance [3, 6, 9, 11] and data relative to the Australian setting are limited [7]. Lastly, most of the large studies on this topic are from the last decades [2, 5, 6, 9] and data on the most recent years are lacking despite a rapidly changing epidemiology over time has been previously reported [4, 5] and the need of constantly updating the knowledge in this field has proven to be essential. Therefore, aim of this study was investigate the epidemiology of BSI among patients with haematological malignancies in Queensland, Australia, over the last 20 years and explore factors associated with clinical outcomes. The present study will improve the current knowledge in the field by providing a multicentric state-wide epidemiological overview over a 20-year timeframe in a geographical setting with a unique prevalence of antimicrobial resistance [12] highlighting the differences between neutropenic and non-neutropenic patients.

## Methods

All episodes of BSI diagnosed by the by the state-wide microbiology service of Pathology Queensland in adult patients with haematological malignancies between January 1, 2000, and December 31, 2019, were included in the study. Patients with haematological malignancies were selected and further classified as neutropenic at the time of the BSI, based on ICD-10 codes (supplementary table 1).

### Definitions

An episode of BSI was defined as a positive blood culture that sustained growth other than skin contaminants or the growth of potential skin contaminants in at least 2 consecutive blood cultures [13]. Isolates of the same species required to be 30 days apart to be classified as a new BSI episode.

BSI episodes were classified as having hospital-onset if the index blood culture was obtained 2 days after hospital admission or within 2 days of discharge; if the index blood culture was collected within the first 2 days of hospital stay BSI were classified as community-associated or healthcare-associated according to Friedman et al. [14]. When one or more organisms were co-isolated within a 48-h period, the BSI was classified as polymicrobial. For data analysis, pathogens were grouped according to main genera and species; Amp-C producers including *Enterobacter* spp., *Serratia* *marcescens*, *Citrobacter* *freundii,* *Providencia* spp. and *Morganella* *morganii* were referred to as “ESCPM” [15, 16]. The presence of a focal/localized infection associated to the BSI was determined using a combination of major and primary diagnostic codes. Haematological malignancies were classified according to the WHO guidelines [17, 18].

### Microbiology

Methods used for species identification over the study period included the VITEK 1, API20E, API20NE (bioMérieux), and Microscan prior to 2008, and the VITEK 2 and VITEK-MS (bioMérieux) later in the study period. Specifically, matrix-assisted laser desorption ionization–time of flight mass spectrometry (MALDI-TOF MS) was introduced in 2012. Antibiotic susceptibility testing was performed using disc diffusion and automated broth microdilution on the VITEK 2, according to recognized standards at the time of testing (CLSI or EUCAST).

### Statistical analysis

Statistical analysis was carried out with Stata 17 (StataCorp, College Station, TX, USA). Categorical variables are presented as frequency and proportion (%), continuous variables as median and interquartile range (IQR). Chi-squared and Fisher exact tests were used to compare variables between groups. A multivariable logistic regression analysis including the first BSI episode per patient was used to determine factors associated with 30-day case-fatality. All relevant variables based on clinical knowledge were included in the model. P values < 0.05 were deemed to represent statistical significance. Kaplan–Meier curves were built for 30-day case-fatality according to neutropenic status.

### Ethics approval

Approval was granted to this study by the Royal Brisbane and Women’s Hospital ethics committee, with a waiver of patient consent (LNR/2020/QRBW/62494).

## Results

We identified 7749 incident BSI in 5159 patients in the study period, of which 4503 (58%) were associated with neutropenia. 3570 patients (69.2%) had a single episode of BSI, 994 (19.3%) had 2 episodes, 354 (6.9%) had 3 episodes and the remaining 241 patients (4.7%) had 4 or more.

### Clinical characteristics

The clinical characteristics of the BSI episodes are summarised in Table 1. Significant differences were noted according to neutropenic status, with BSI during neutropenia being characterised by a younger median patient age (*p* < 0.001), lower Charlson Index (*p* < 0.001) and lower 30-day case-fatality (*p* < 0.001). Differences between the two groups were also noted in the underlying malignancies and in the mode of acquisition, with BSI in neutropenic patients being more likely to be hospital-acquired (57.3% vs 37%, *p* < 0.001).

**Table 1:** Clinical characteristics of BSI episodes, overall and according to neutropenic status

	Total	Neutropenia	Non-neutropenia	*p* value
	N = 7749	N = 4503	N = 3246	
Male sex	4694 (60.5%)	2705 (60.1%)	1985 (61.2%)	0.33
Age	62.7 (50.5–71.7)	59.9 (47.7–68.4)	66.9 (54.6–76.5)	< 0.001
Charlson Comorbidity Index	3 (2–4)	2 (2–4)	3 (2–5)	< 0.001
Haematological malignancy				
MPN neoplasms	320 (4.1%)	46 (1.0%)	274 (8.4%)	< 0.001
Mastocytosis	2 (0.0%)	1 (0.0%)	1 (0.0%)	0.82
MDS/MPN neoplasms	60 (0.8%)	25 (0.6%)	35 (1.1%)	0.010
MDS syndromes	385 (5.0%)	125 (2.8%)	260 (8.0%)	< 0.001
AML and related neoplasms	2,311 (29.8%)	1,818 (40.4%)	493 (15.2%)	< 0.001
Acute leukemias of ambiguous lineage	50 (0.6%)	40 (0.9%)	10 (0.3%)	0.002
Lymphoblastic leukemia	643 (8.3%)	494 (11.0%)	149 (4.6%)	< 0.001
Mature B-cell neoplasms	3,461 (44.6%)	1,674 (37.2%)	1,787 (55.0%)	< 0.001
Hodgkin lymphoma	150 (1.9%)	86 (1.9%)	64 (2.0%)	0.85
Mature T and NK neoplasms	238 (3.1%)	139 (3.1%)	99 (3.0%)	0.93
Histiocytic and dendritic cell neoplasms	6 (0.1%)	4 (0.1%)	2 (0.1%)	0.67
Other leukemias, non-classified	41 (0.5%)	10 (0.2%)	31 (1.0%)	< 0.001
Other neoplasms of lymphoid, hematopoietic & related tissue, unspecified	82 (1.1%)	41 (0.9%)	41 (1.3%)	0.13
Focal/localised infection identified				
No	6,583 (85%)	4,209 (93.5%)	2,374 (73.1%)	< 0.001
Yes	1166 (15%)	294 (6.5%)	872 (26.9%)	< 0.001
Polymicrobial	1021 (13.2%)	673 (14.9%)	348 (10.7%)	< 0.001
Mode of acquisition				
Hospital acquired	3,782 (48.8%)	2,582 (57.3%)	1,200 (37.0%)	< 0.001
Healthcare-associated	3,238 (41.8%)	1,747 (38.8%)	1,491 (45.9%)	< 0.001
Community acquired	729 (9.4%)	174 (3.9%)	555 (17.1%)	< 0.001
Length of stay	20 (9–36)	24 (11–37)	15 (7–32)	< 0.001
Day-30 case fatality	1,543 (19.9%)	755 (16.8%)	788 (24.3%)	< 0.001

Data are presented as median (IQR) for continuous measures, and n (%) for categorical measures

*BSI* Bloodstream infection, *MPN* myeloproliferative, *MDS* Myelodysplastic; *MDS*/*MPN* myelodysplastic/myeloproliferative neoplasms, *AML* Acute myeloid leukemia

### Causative pathogens

Out of 7749 incident infection episodes, 1021 (13.2%) were polymicrobial and overall included 8987 microbial isolates. BSI causative pathogens are summarised in Table 2 according to neutropenic status. Overall, Gram-negatives were responsible of most BSI episodes (58%) and were more common in neutropenic than non-neutropenic patients (*p* < 0.001). The most common isolates were *E.* *coli* (15.4%), followed by *Pseudomonas* spp. (14.2%), coagulase-negative staphylococci (CoNS) (10.5%) and *Staphylococcus* *aureus* (9.8%).

**Table 2:** Causative pathogens of BSI, overall and according to neutropenic status

	Total	Neutropenia	Non-neutropenia	p-value
	N = 8987	N = 5309	N = 3678	
Gram-negatives	5240 (58.3%)	3308 (62.3%)	1932 (52.5%)	< 0.001
*E.* *coli*	1,385 (15.4%)	855 (16.1%)	530 (14.4%)	0.027
*Klebsiella* spp.	866 (9.6%)	617 (11.6%)	249 (6.8%)	< 0.001
ESCMP	694 (7.7%)	434 (8.2%)	260 (7.1%)	0.052
Other *Enterobacterales*^a^	142 (1.6%)	72 (1.4%)	70 (1.9%)	0.041
*Pseudomonas* spp.	1,273 (14.2%)	879 (16.6%)	394 (10.7%)	< 0.001
*Acinetobacter* spp.	166 (1.8%)	88 (1.7%)	78 (2.1%)	0.11
*S.* *maltophilia*	238 (2.6%)	146 (2.7%)	92 (2.5%)	0.47
*Achromobacter* spp.	29 (0.3%)	14 (0.3%)	15 (0.4%)	0.24
*Aeromonas* spp.	43 (0.5%)	27 (0.5%)	16 (0.4%)	0.62
*Burkholderia* spp.	26 (0.3%)	14 (0.3%)	12 (0.3%)	0.59
*Cepacia* *complex*	12 (0.1%)	6 (0.1%)	6 (0.2%)	0.52
*Pseudomallei*	10 (0.1%)	5 (0.1%)	5 (0.1%)	0.56
Other spp.	4 (0.0%)	3 (0.1%)	1 (0.0%)	0.52
*Capnocytophaga* spp.	37 (0.4%)	31 (0.6%)	6 (0.2%)	0.002
*Chryseobacterium/E.meningoseptica/Sphingobacterium* sp	32 (0.4%)	13 (0.2%)	19 (0.5%)	0.034
*Haemophilus* spp.	53 (0.6%)	13 (0.2%)	40 (1.1%)	< 0.001
*Moraxella* spp.	25 (0.3%)	11 (0.2%)	14 (0.4%)	0.13
*Sphingomonas* spp.	41 (0.5%)	24 (0.5%)	17 (0.5%)	0.95
*Salmonella* spp.	43 (0.5%)	9 (0.2%)	34 (0.9%)	< 0.001
Other Gram-negatives^b^	147 (1.6%)	61 (1.1%)	86 (2.3%)	< 0.001
Gram-positives	3,202 (35.6%)	1,702 (32.0%)	1,500 (40.7%)	< 0.001
*S.* *aureus*	885 (9.8%)	312 (5.9%)	573 (15.6%)	< 0.001
Coagulase negative *Staphylococci*	946 (10.5%)	603 (11.4%)	343 (9.3%)	0.002
*E.* *faecalis*	233 (2.6%)	122 (2.3%)	111 (3.0%)	0.035
*E.* *faecium*	161 (1.8%)	128 (2.4%)	33 (0.9%)	< 0.001
*Streptococcus* spp.				
Viridans Group *Streptococci*	280 (3.1%)	234 (4.4%)	46 (1.2%)	< 0.001
*S.* *pneumoniae*	191 (2.1%)	31 (0.6%)	160 (4.3%)	< 0.001
Beta-haemolytic *Streptococci*	114 (1.3%)	41 (0.8%)	73 (2.0%)	< 0.001
*S.* *bovis* group	36 (0.4%)	22 (0.4%)	14 (0.4%)	0.80
Other *Streptococcus* spp.	25 (0.3%)	17 (0.3%)	8 (0.2%)	0.36
*Bacillus* spp.	40 (0.4%)	24 (0.5%)	16 (0.4%)	0.90
*Corynebacterium* spp.	57 (0.6%)	39 (0.7%)	18 (0.5%)	0.15
*Gemella* spp.	23 (0.3%)	20 (0.4%)	3 (0.1%)	0.006
*L.* *monocytogenes*	23 (0.3%)	7 (0.1%)	16 (0.4%)	0.005
Other Gram-positives^c^	188 (2.1%)	102 (1.9%)	86 (2.3%)	0.18
Anaerobes Gram-negative	136 (1.5%)	56 (1.1%)	80 (2.2%)	< 0.001
Anaerobes Gram-positive	126 (1.4%)	64 (1.2%)	62 (1.7%)	0.058
Mycobacteria	36 (0.4%)	22 (0.4%)	14 (0.4%)	0.80
Yeasts				
Candida spp.^d^	185 (2.1%)	115 (2.2%)	70 (1.9%)	0.38
Other yeasts (non-Candida)^e^	28 (0.3%)	14 (0.3%)	14 (0.4%)	0.33
Moulds^f^	34 (0.4%)	28 (0.5%)	6 (0.2%)	0.006

^a^Including *Citrobacter* spp. (other than *C.* *freundii*)., Coliform spp., *Escherichia* spp. (other than *E.* *coli)*, *Proteus* spp., *Serratia* spp. (other than *S.* *marcescens*), *Pantoea* spp., *Edwardsiella* spp., *Hafnia* spp., *Kluyvera* spp., *Leclercia* spp., *Rahnella* spp., *Raoultella* spp., *Yersinia* spp

^b^Including *Agrobacterium* spp., *Alcaligenes* spp., *Arcobacter* spp., *Bordetella* spp., *Brevundimonas* spp., *Campylobacter* spp., *Chromobacterium* spp., *Comamonas* spp., *Delftia* spp., *Helicobacter* spp., *Methylobacterium* spp., *Neisseria* spp., *Ochrobactrum* spp., *Pandoraea* spp., *Pasteurella* spp., *Ralstonia* spp., *Rhizobium* spp., *Roseomonas* spp., *Shewanella* spp., *Vibrio* spp., *Eikenella* spp., *Kingella* spp

^c^Including *Aerococcus* spp., *Alloiococcus* spp., *Arthrobacter* spp., *Brevibacterium* spp., *Cellulomonas* spp., *Dermabacter* spp., *Erysipelothrix* spp., *Enterococcus* spp. (other than *E.* *faecalis* and *faecium*), *Kocuria* spp., *Lactobacillus* spp., *Lactococcus* spp., *Leuconostoc* spp., *Microbacterium* spp., *Nocardia* spp., *Oerskovia* spp., *Paenibacillus* spp., *Pediococcus* spp., *Rhodococcus* spp., *Rothia* spp., S*.* *lugdunensis,* *Abiotrophia* spp., *Granulicatella* spp., *Actinomyces* spp

^d^Including *C.* *albicans* (n = 62), *C.* *glabrata* (n = 35) *C.* *parapsilosis* (n = 29), *C.* *tropicalis* (n = 21), *C.* *krusei* (n = 20), *C.* *dubliniensis* (n = 2*),* *C.* *lusitaniae* (n = 2), *C.* *kefyr* (n = 2), *C.* *guilliermondii* (n = 2), *C.* *famata* (n = 1), unspeciated (n = 9). ^e^Including *Cryptococcus* spp. (n = 16), *Trichosporon* spp. (n = 8), *Rhodotorula* spp. (n = 2), *S.* *cerevisiae* (n = 2). ^f^ Including *Scedosporium* spp. (n = 18), *Fusarium* spp. (n = 12), *Aspergillus* spp. (n = 1), *Penicillium* sp. (n = 1), unspeciated (n = 2)

Differences were noted in the prevalence of causative pathogens according to neutropenic status the most significant of which were relative to *Pseudomonas* spp. (*p* < 0.001), *Klebsiella* spp. (*p* < 0.001), *Enterococcus* *faecium* (*p* < 0.001), viridans-group streptococci (*p* < 0.001) and moulds (*p* = 0.003) that were more common in neutropenic than non-neutropenic patients, while *S.* *aureus* was more frequent in non-neutropenic patients (*p* < 0.001).

### Antimicrobial resistance patterns

Antimicrobial susceptibility testing was available for most isolates and antimicrobial resistance rates are summarised in supplementary table 2A-B. Resistance to 3rd generation cephalosporins was found in 5.8% of *Klebsiella* spp., 8.2% of *Escherichia* *coli* and 27.5% of ESCPM. 7% of Enterobacterales (202/2832 isolates) were also resistant to ciprofloxacin while carbapenem resistance was below 2%. 18.6% of *S.* *aureus* strains were resistant to anti-staphylococcal penicillins (methicillin-resistant *S.* *aureus*, MRSA) and 32.4% of *E.* *faecium* showed vancomycin-resistance. 26.9% of viridans-group streptococci were penicillin-resistant.

Differences were noted in the prevalence of antimicrobial resistance according to neutropenic status (Supplementary table 3), with higher prevalence found in neutropenic than non-neutropenic patients for several antimicrobials including *E.* *coli* resistance to ampicillin (59.2% vs 52%, *p* = 0.016), amoxicillin-clavulanate (24.9% vs 19.1% *p* = 0.018), ciprofloxacin (12.4% vs 8.3%, *p* = 0.021) and piperacillin-tazobactam (9.2% vs 5.7%, *p* = 0.050). Resistance to trimethoprim-sulfamethoxazole was also more frequent in neutropenic than non-neutropenic patients both in *E.* *coli* (47.9% vs 36.9%, *p* < 0.001), ESCPM (30.8% vs 17.4%, *p* < 0.001) and *Stenotrophomonas* *maltophilia* (8% vs 1.3% *p* = 0.038). Penicillin-resistant streptococci (21.6% vs 10.8%, *p* < 0.001) and vancomycin-resistant enterococci (18.4% vs 6.3%, *p* < 0.001) were also more frequent during neutropenia.

### Trends over time

**Fig. 1:**
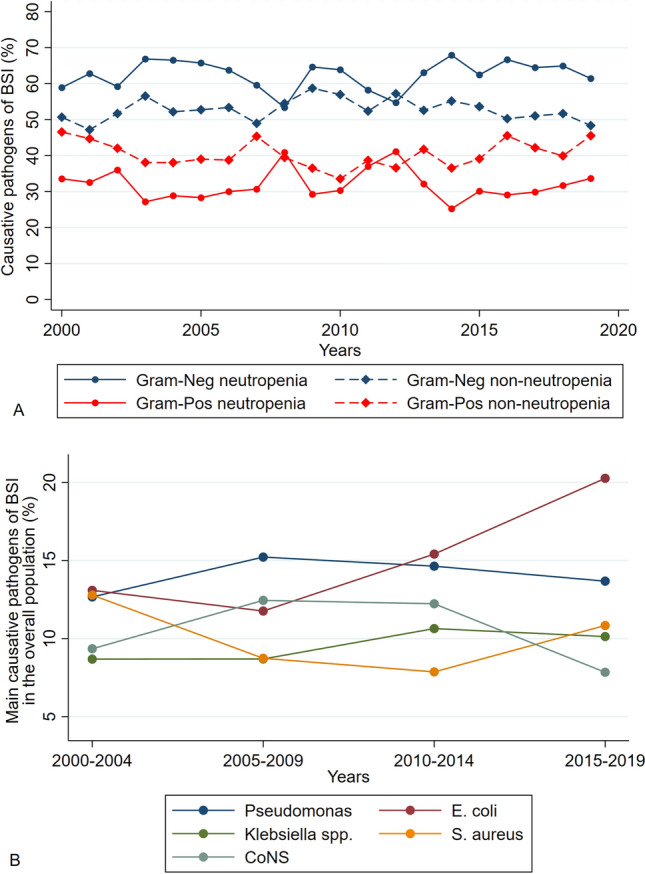
Trends causative BSI pathogens over time. **a** Trends of Gram-positives and Gram-negatives over time according to neutropenic status. **B **Trends of the 5 main causative pathogens over time in the whole population. *CoNS* Coagulase-Negative Staphylococci

Gram-negatives remained the main BSI causative pathogens throughout the whole study period (Fig. 1a). *E.* *coli,* *Pseudomonas* spp., *S.* *aureus,* CoNS and *Klebsiella* spp. were the 5 most common pathogens throughout the whole study period with rates of *E.* *coli* showing an increasing trend from 13.1% (217/1658 isolates) in 2000–2004 to 20.3% (514/2537) in 2015–2019 (*p* < 0.001), overcoming rates of *Pseudomonas* spp. from 2012 onwards. Figure 1b shows the trends of the 5 most common causative pathogens over time.

**Fig. 2:**
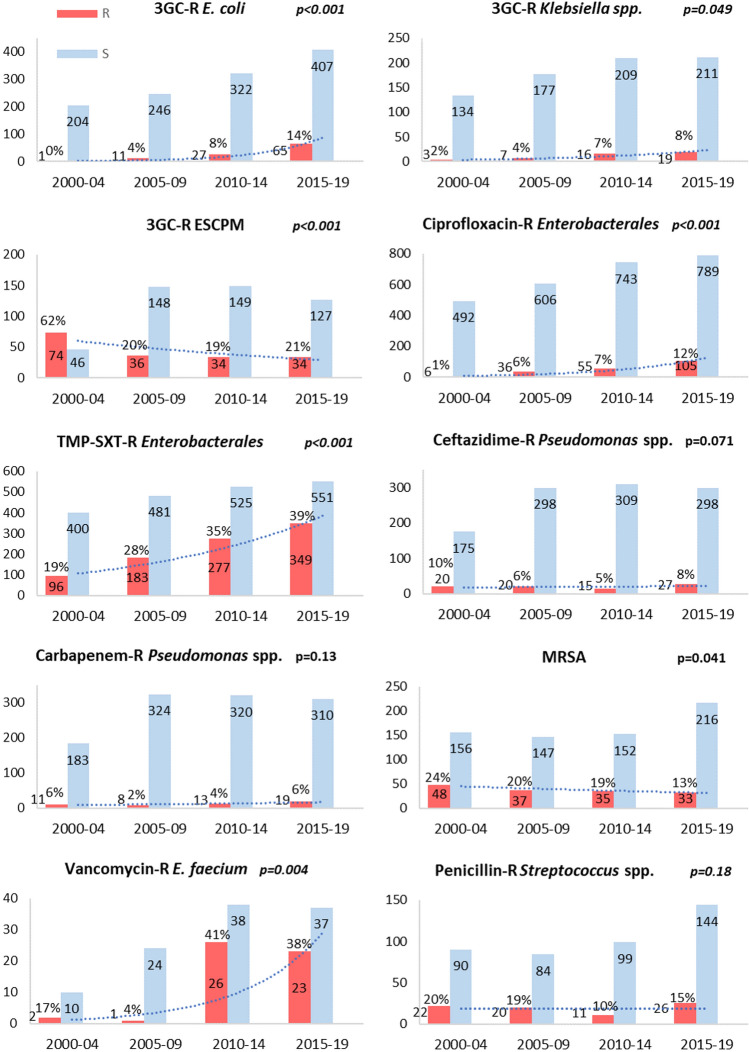
Resistance rates of BSI isolates to main antimicrobial classes over time. *3GC* 3rd Generation Cephalosporins, *R* resistant, *ESCPM* *Enterobacter* spp., *Serratia marcescens*, *Citrobacter freundii*, *Providencia* spp. and *Morganella morganii*, *TMP-SXT* Trimethoprim-sulfamethoxazole, *MRSA* methicillin-resistant *S. aureus*

Trends of antimicrobial resistance showed significant changes over time (Fig. 2): increasing resistance trends were noted in Enterobacterales for ciprofloxacin (*p* < 0.001) and trimethoprim-sulfamethoxazole (*p* < 0.001) as well as for 3^rd^ generation cephalosporin (*E.* *coli,* *p* < 0.001; *Klebsiella* spp. *p* = 0.049) with the exception of ESCPM where the trend was decreasing (*p* < 0.001). Rate of MRSA decreased over time (*p* = 0.041) while rates of vancomycin-resistant *E.* *faecium* significantly increased (*p* = 0.004).

These temporal trends were confirmed both in neutropenic and non-neutropenic patients (data not shown) with the most consistent change over time observed for trimethoprim-sulfamethoxazole-resistant Enterobacterales in neutropenic patients (from 20.7%, 57/276 isolates in 2000–2004 to 43.2%, 240/556 isolates in 2015–2019, difference: 22.5%, 95%CI 15–28%, *p* < 0.001).

### Case-fatality

**Fig. 3:**
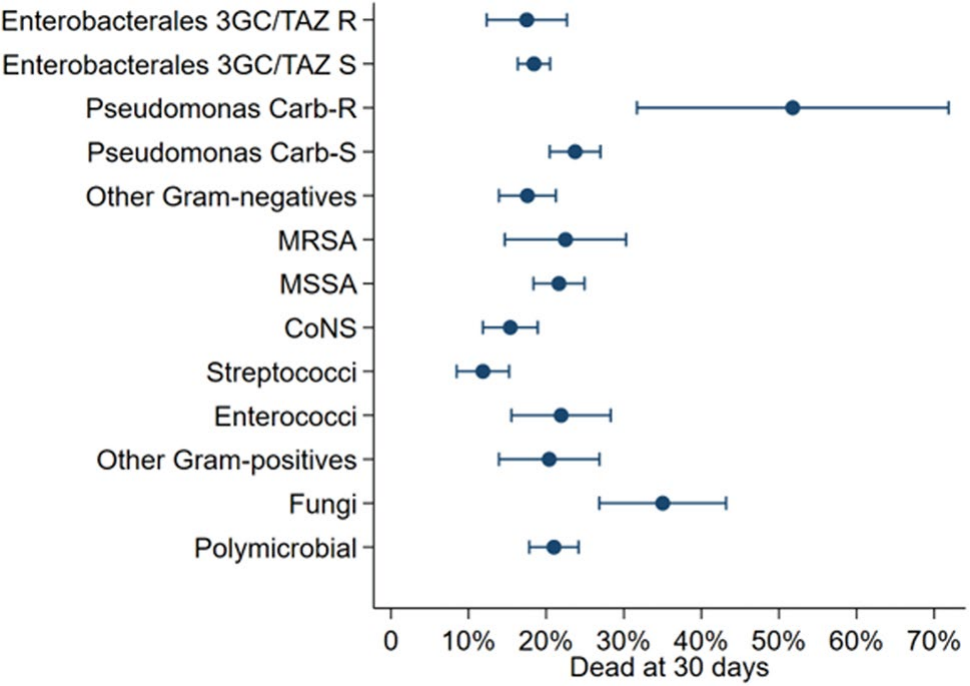
30-day fatality rate (%) with 95%CI for the different groups of causative pathogens, adjusted for the covariates in Table 3. *3GC* 3rd Gen Cephalosporins, *TAZ* Piperacillin-Tazobactam, *Carb* Carbapenem, *R* resistant, *S* susceptible

Factors associated with 30-day case-fatality were assessed considering the first BSI episode per patient. Causative pathogens were analysed according to main species or genera and susceptibility to the main antimicrobial classes. Univariate analysis for 30-day case-fatality is shown in Table 3. All variables were also included in a multivariable logistic regression model (also in Table 3). Figure 3 shows the marginal 30-day case-fatality rate for the different groups of pathogens, adjusted for any covariates in the model. Supplementary Fig. 3 shows Kaplan–Meier curves according to neutropenic status.

**Table 3:** Univariate and multivariate analysis of factors associated with 30-day case-fatality for first BSI episode

	Univariate analysis		Multivariate analysis	
	Dead at day-30	*p* value	OR	95%CI
	N = 1005/5159 (19.5%)			
Neutropenic status		** < 0.001**		
Non-neutropenia	576/2325 (24.8%)		1*	
Neutropenia	421/2784 (15.1%)		**0.66**	**0.55 to 0.78**
Gender		0.33		
Female	367/1958 (18.7%)		1*	
Male	629/3146 (20.0%)		1.0	0.86 to 1.17
Age		** < 0.001**	**1.43**^§^	**to 1.52**
< 50	101/1,076 (9.4%)			
50–59	96/877 (10.9%)			
60–69	254/1,379 (18.4%)			
70–79	311/1,140 (27.3%)			
80–89	190/545 (34.8%)			
> = 90	44/88 (50.0%)			
Charlson Index		** < 0.001**		
< = 2	307/2,474 (12.4%)		1*	
3–4	334/1,486 (22.5%)		**1.72**	**to 2.06**
> = 5	355/1,145 (31.0%)		**2.41**	**2.0 to 2.91**
Underlying malignancy		** < 0.001**		
Acute leukemias (AML, ALL, ambiguous lin.)	230/1,589 (14.5%)		1.05	0.87 to 1.28
MDS & MPN neoplasms	141/587 (24.0%)		1.10	0.588 to 1.39
Lymphomas (Mature B/T/NK & LH)	600/2,825 (21.2%)		1*	
Other neoplasms	25/104 (24.0%)		1.15	0.71 to 1.87
Focal/localized infection identified		0.26		
No	814/4,232 (19.2%)		**1.36**	**1.11 to 1.67**
Yes	182/873 (20.8%)		1*	
Mode of acquisition		0.20		
Hospital-acquired	483/2,363 (20.4%)		**1.33**	1.03 **to 1.73**
Healthcare-associated	404/2,127 (19.0%)		1.05	0.81 to 1.36
Community-acquired	109/615 (17.7%)		1*	
Causative pathogen		** < 0.001**		
*Monomicrobial* *infection*				
Gram-negatives				
*Enterobacterales*	286/1,543 (18.5%)			
3GC/TAZ R	34/187 (18.2%)		1.18	0.73 to 1.92
3GC/TAZ S	239/1,244 (19.2%)		1.27	0.91 to 1.76
*Psudomonas* spp.	154/635 (24.3%)			
Carbapenem-R	9/20 (45.0%)		**7.32**	**2.78 to 19.32**
Carbapenem-S	140/589 (23.8%)		**1.80**	**1.26 to 2.57**
Other Gram-negatives	65/416 (15.6%)		1.19	0.80 to 1.78
Gram positives				
*S.* *aureus*	165/629 (26.2%)			
MRSA	25/87 (28.7%)		1.67	0.94 to 2.99
MSSA	133/504 (26.4%)		**1.58**	**1.10 to 2.27**
Coagulase-negative *Staphylococci*	56/431 (13.0%)		1*	
*Streptococcus* spp.	39/356 (11.0%)		0.72	0.46 to 1.14
*Enterococcus* spp.	34/135 (25.2%)		1.61	0.97 to 2.66
Other Gram-positives	28/137 (20.4%)		1.45	0.86 to 2.46
Fungal organisms (yeasts and moulds)	42/110 (38.2%)		**3.33**	**2.02 to 5.48**
Mycobacteria	0/6 (0.0%)			
Polymicrobial infection	119/628 (18.9%)		**1.51**	**1.06 to 2.16**

Data are presented as n/total (row %). *AML* Acute Myeloid Leukaemia, *ALL* Acute Lymphoblastic Leukaemia, *MDS* Myelodysplastic Syndromes, *MPN* Myeloproliferative Neoplasms, *LH* Hodgkin Lymphoma, *3GC* 3rd Gen Cephalosporins; *TAZ* Piperacillin-Tazobactam, *R* Resistant; *S* Susceptible, *MRSA* Methicillin-Resistant *S.* *aureus*, *MSSA* Methicillin Susceptible *S.* *aureus.* 1* = reference category; ^§^per 10y age increase

## Discussion

To our knowledge this is one of the largest studies reporting the epidemiology of BSI in haematology patients in the last decades. The approach of using a state-wide microbiology service for the selection of the BSI episodes has allowed a comprehensive and reliable mapping of the causative pathogens and associated resistance patterns over time.

The study has confirmed the recent shift reported in the literature towards Gram-negatives as main BSI determinants in haematology patients also in our geographical area [2, 5, 6], and has highlighted how significant differences exist in the epidemiology of BSI between neutropenic and non-neutropenic patients. These include a higher prevalence of Gram-negatives, in particular *Pseudomonas* and *Klebsiella* spp., in neutropenic compared to non-neutropenic patients, as well as higher rates of enterococci and viridans-group streptococci, likely explained by the role of chemotherapy-induced mucositis as a key risk factor for BSI during neutropenia [6, 19].

Interestingly, neutropenic patients suffered from lower rates of *S.* *aureus* BSI compared to non-neutropenic patients, and this could be explained by their lack of ability to mount an adequate immune response, preventing the metastatic dissemination typical of *S.* *aureus* bacteraemia [20–22]. Despite the impact of neutropenia on the risk of developing *S.* *aureus* bacteraemia has not been well established yet [20–22], previous studies have shown how *S.* *aureus* bacteraemia in neutropenic patients is more often acquired without known portal/primary focus of infection and less frequently results in osteomyelitis or endocarditis compared to non-neutropenic patients [22]. These clinical features have also sometimes been associated to lower mortality rates in neutropenic versus non-neutropenic patients [20]. As discussed by Camp et al. in their recent paper, the persistence of *S.* *aureus* inside phagocytes can facilitate its dissemination into metastatic infection, and recent animal models have shown that these intracellular reservoirs of *S.* *aureus*, acting as Trojan horses, are associated with significant virulence even in the presence of antibiotics [22, 23]. Data from South Australia about the epidemiology of BSI in neutropenic patients also highlighted a very low prevalence of bacteraemia due to *S.* *aureus*, overall accounting for less than 4% of the isolates of the entire cohort [7].

Yet in a geographical area with a low-prevalence of antimicrobial resistance such as Australia [12], we observed a higher prevalence of resistance in neutropenic than non-neutropenic patients, possibly due to their higher exposure to antimicrobials, in the setting of recurrent febrile episodes. Our data also show a progressive increase of resistance over time, more consistent in neutropenic than non-neutropenic patients, of which the most significant increase is the one observed for vancomycin-resistant enterococci, in line with what reported by Carvahlo et al. [7] in neutropenic patients in South Australia and, consistent with the overall national trend [24]. An exception to the increasing resistance trends over time in our cohort is represented by the rate of MRSA, and resistance to 3^rd^ generation cephalosporins in ESCPM bacteria, both decreasing over time. A decrease in the rates of hospital-acquired MRSA in the last decades has been previously reported in Australia [25] and is in line with multiple U.S. and European cohorts [26]. Among factors that may have played a role in this reduction are improvements in the management of invasive devices, national reporting of *S.* *aureus* bacteraemia, and the National Hand Hygiene Initiative [25, 27]. Conversely, the high prevalence of resistance to 3^rd^ generation cephalosporins in ESCPM bacteria in 2000–2004 in our cohort was unexpected, and the reasons behind it not fully clear. We could possibly speculate a widespread use of ceftazidime before the implementation of Antimicrobial Stewardship Programs (ASP), responsible for a significant rate of AmpC-mutants derepression during those years [28]. Another consideration about the antimicrobial resistance patterns we observed pertains to their association with case fatality. To this respect,

it is significant to note how carbapenem-resistant *Pseudomonas* spp. was associated to the highest odds of case-fatality at multivariate analysis, highlighting the threat that resistance to last-resort antibiotics poses to human health [12].

Neutropenia and more in general immunosuppression, confers an additional risk of adverse outcomes during BSI, compared to immunocompetent patients [29]. However, the role of neutropenia as a predictor of mortality in patients with haematological malignancies and BSI is more complex as several other factors also take part in the risk stratification of patients in this context, including the duration and severity of the neutropenia itself [3], the stage and type of the underlying malignancy [30], the overall risk assessment of serious complications based on validated scoring systems [31], as well as the presence of extensive tissue involvement by the infection [32, 33]. In this context, in cohorts of patients with haematological malignancies and BSI, neutropenia might not be a major determinant of short-term mortality anymore, particularly in critically ills [33, 34]. Indeed, BSI during neutropenia might have similar [11, 35] or even sometimes better outcomes [10, 20, 36] compared to those in non-neutropenic patients, in particular if neutropenia is of short duration [3] and in the absence of metastatic localisation of infection, but rather in the context of transient bacteraemia [20, 21, 32]. BSI in neutropenic patients may also have better or comparable outcomes when compared to BSI in highly comorbid non-neutropenic patients [10, 20, 21]. In our study neutropenia was independently associated with survival and this could be due to the higher burden of comorbidities and older age of non-neutropenic patients influencing overall survival. Unfortunately, we were unable to stratify outcomes according to the duration of the neutropenia nor adjust them based on a risk assessment of serious complications [31].

We acknowledge our study has several limitations. The first limitation is the retrospective nature of the study design, limiting the generalisability of our conclusions in particular relatively to case fatality. Within the context of the retrospective study design, our results are further limited by the lack of clinical data about antimicrobial treatment and prophylaxis as well as about haemato-oncological regimens (including bone marrow transplantation), the malignancy stage, performance and comorbidity scores specifically validated in cancer patients [37], the duration and severity of the neutropenia [3], and the clinical severity at BSI presentation [38], which would be all relevant factors to assess in relation with antimicrobial resistance trends and more importantly clinical outcomes. Even among available data, limitations should be acknowledged. Indeed, the use of ICD-10 codes to select and classify patients was not cross-validated and might have been subject to administrative classification errors. Similarly, the use of ICD-10 codes to classify patients in neutropenic and non-neutropenic (in the absence of a validation via review of laboratory tests) prevented a precise temporal association between the BSI onset and the low neutrophil counts. The identification of focal/localised infections associated to the BSI was also crude, based on a combination of major diagnostic codes and not on a case-by-case review and detailed information about the BSI source was not available.

Relatively to our methodology, a limitation pertains to our definition of BSI, according to which contaminants were excluded based on the number of positive BC sets but in the absence of data about (semi)-quantitative line-tip culture in cases suspected to be central line associated [39]. This might have led us to include as true infections some cases that were instead contaminations, in particular when positive BC sets were taken from the central line only and not from the peripheral vein, making it difficult to distinguish between true BSI and BC contamination. Overall, however the rate of BSI due to CoNS we reported was lower compared to several other studies in similar settings [9]. It should also be acknowledged that not all isolates were tested against a standard unified method and that the testing, reporting and interpretation of the antimicrobial susceptibility testing has evolved over time. In this context, the susceptibility profiles reported in the study may have been influenced by the changes recommended by CLSI to the breakpoints of some antimicrobials over time [40], as well as by the change from CLSI to EUCAST methodologies occurring in Queensland during the study period [41]. Lastly, with respect to the statistical analysis, it should be acknowledged that no correction was made for multiple testing.

In conclusion, our data confirm the shift reported in the last 20 years from Gram-positives to Gram-negatives as main determinants of BSI in patients with haematological malignancies. An increase in antimicrobial resistance was observed in our cohort, especially in neutropenic patients. Overall, these epidemiological trends warrant close monitoring, in order to appropriately inform the management of antimicrobial prophylaxis and empirical treatment protocols.

### Supplementary Information

Below is the link to the electronic supplementary material.

Supplementary file 1 (DOCX 72 KB)
