# Supplementary material for: Bloodstream infections in neutropenic and non-neutropenic patients with haematological malignancies: epidemiological trends and clinical outcomes in Queensland, Australia over the last 20 years
Source: Clin Exp Med. 2023 Oct 10;23(8):4563–73. doi: 10.1007/s10238-023-01206-x (PMC10725384; doi:10.1007/s10238-023-01206-x)

**Supplementary material**

**Supplementary table 1. ICD-10 codes used in this manuscript for patients’ selection and classification**

| **Primary haematological malignancy diagnosis** | **ICD-10** |
| --- | --- |
| Hodgkin lymphoma | C81, including C81.0, C81.1, C81.2, C81.3, C81.4, C81.7, C81.9 |
| Follicular lymphoma | C82, including C82.0, C82.1 C82.2, C82.3, C82.4, C82.5, C82.6, C82.7, C82.9 |
| Non-follicular lymphoma | C83, including C83.0, C83.1, C83.3, C83.5, C83.7, C83.8, C83.9 |
| Mature T/NK-cell lymphomas | C84, including C84.0, C84.1, C84.4, C84.5, C84.6, C84.7, C84.8, C84.9 |
| Other and unspecified types of non-Hodgkin lymphoma | C85, including C85.1, C85.2, C85.7, C85.9 |
| Other specified types of T/NK-cell lymphoma | C86, including C86.0, C86.1, C86.2, C86.3, C86.4, C86.5, C86.6 |
| Malignant immunoproliferative diseases | C88, including C88.0, C88.2, C88.3, C88.4, C88.7, C88.9 |
| Multiple myeloma and malignant plasma cell neoplasms | C90, including C90.0, C90.1, C90.2, C90.3 |
| Lymphoid leukaemia | C91, including C91.0, C91.1, C91.3, C91.4, C91.5, C91.6, C91.7, C91.8, C91.9 |
| Myeloid leukaemia | C92, including C92.0, C92.1, C92.2, C92.3, C92.4, C92.5, C92.6, C92.7, C92.8, C92.9 |
| Monocytic leukaemia | C93, including C93.0, C93.1, C93.3, C93.7, C93.9 |
| Other leukaemias of specified cell type | C94, including C94.0, C94.2, C94.3, C94.4, C94.6, C94.7 |
| Leukaemia of unspecified cell type | C95, including C95.0, C95.1, C95.7, C95.9 |
| Other and unspecified malignant neoplasms of lymphoid, haematopoietic, and related tissue | C96, including C96.0, C96.2, 96.4, 96.5, 96.6, 96.7, 96.8, 96.9 |
| **Other diagnosis of hospitalization** |  |
| Agranulocytosis | D70 |

-

**Supplementary table 2a. Antimicrobial susceptibility** **profiles of Gram-negative isolates**

|  | **AMP** | **AUG** | **3GC^a^** | **CAZ** | **FEP** | **TAZ** | **MER/IPM** | **CIP** | **GEN** | **AK** | **TMP-SXT** |
| --- | --- | --- | --- | --- | --- | --- | --- | --- | --- | --- | --- |
| ***E. coli*** | 43.5%  (561/1288) | 77.4%  (988/1277) | 91.9%  (1181/1285) | - | - | 92.1%  (926/1005) | 100%^b^  (1257/1257) | 89.2%  (1128/1264) | 92.9%  (1197/1288) | 99.2%  (1256/1266) | 56.5%  (724/1281) |
| ***Klebsiella* spp.** | - | 90.4%  (699/773) | 94.2%  (731/776) | - | - | 90.24%  (564/625) | 99.9%^b^  (763/764) | 95.6%  (733/767) | 96.4%  (749/777) | 99.9%  (766/767) | 78.04%  (604/774) |
| **ESCPM** | - | - | 72.5%  (470/648) | - | - | 84.2%  (420/499) | 98.7%^b^  (633/641) | 95.2%  (609/640) | 92.3%  (601/651) | 99.4%  (636/640) | 74.3%  (480/646) |
| ***Pseudomonas* spp.** | - | - | - | 92.94%  (1080/1162) | 95.6%  (912/954) | 91.2% (838/919) | 95.7% (1137/1188) | 95.7% (1143/1195) | 96.8% (1163/1201) | 98.9% (1157/1170) | - |
| ***Acinetobacter* spp.** | - | - | - | - | 83.5% (76/91) | - | 96.3% (132/137) | 97.06% (132/136) | - | 96.85% | - |
| ***S. maltophila*** | - | - | - | - | - | - | - | - | - | - | 94.61% (193/204) |
| ***Achromobacter* spp.** | - | - | - | - | - | - | 100% (28/28) | 57.1% (16/28) | - | - | 92.0% (23/25) |
| ***- B. cepacia* complex**  ***- B. pseudomallei*** | - | - | - | 90.9% (11/11)  100%  (7/7) | 62.5%  (5/8)  25%  (1/4) | -  100%  (3/3) | 72.7% (8/11)  100%  (7/7) | 54.6 %  (6/11)  33%  (2/6) | - | - | 90.9% (10/11)  100%  (7/7) |
| ***Chryseobacterium / E. meningoseptica / Sphingobacterium spp.*** | - | - | - | - | - | - | 32.1% (9/28) | 71.4% (20/28) | - | - | 70.8% (17/24) |
| ***S. paucimobilis*** | 66.7% (16/24) | - | 67.7% (21/31) | - | - | - | 88.2% (30/34) | 67.6% (25/37) | 91.9% (34/37) | 92.3% (24/26) | 80.9% (17/21) |
| ***Aeromonas* spp.** | - | - | 100% (40/40) | - | - | 55%  (11/20) | 73.2%  (30/41) | 97.7% (42/43) | 97.6% (40/41) | 100% (41/41) | 97.6% (42/43) |
| ***Haemophilus* spp.** | 57.1% (28/49) | 91.1% (41/45) | 100% (49/49) | - | - | - |  | 100% (37/37) | - | - | - |
| ***Salmonella*** | 100% (37/37) | 100% (37/37) | 100% (36/36) | - | - | - | 100% (36/36) | 100% (35/35) | 78.4% (29/37) | 77.8% (28/36) | 97.3% (36/37) |

**^a^**Any CRO, CTX, CAZ, FEP; **^b^**For *Enterobacterales* susceptibility to ertapenem is also included. ESCPM = *Enterobacter* spp., *Serratia marcescens*, *Citrobacter freundii, Providencia* spp. and *Morganella morganii.* AMP = Ampicillin; AUG = Amoxicillin-clavulanate; 3GC = 3rd generation cephalosporin; CAZ = Ceftazidime; FEP = Cefepime; TAZ = Piperacillin-Tazobactam; MER = Meropenem; IPM = Imipenem; CIP = Ciprofloxacin; GEN = Gentamicin; AK = Amikacin, TMP-SXT = trimethoprim-sulphametozaxole.

**Supplementary table 2b. Antimicrobial susceptibility profiles of Gram-positive isolates**

|  | **PEN** | **FLU** | **AMP** | **CRO** | **TEC** | **VAN** | **DAP** | **LZD** | **RIF** | **CIP** | **GEN** |
| --- | --- | --- | --- | --- | --- | --- | --- | --- | --- | --- | --- |
| ***S. aureus*** | 13.2% (109/824) | 81.43% (671/824) | - |  | 99.3% (689/694) | - | 100% (417/417) | 99.6% (555/557) | 98.5% (785/797) | 89.2% (710/796) | 89.8% (711/792) |
| **Coagulase Negative *Staphylococci*** | 5%  (39/760) | 14% (112/798) | - |  | 85.1% (616/724) | - | 97.2% (388/399) | 99.8% (614/615) | 90.9% (707/778) | 43.9% (337/767) | 37.8% (296/784) |
| **Viridans *Streptococci*** | 73.1% (133/182) | - | - | 83.6% (46/55) | - | - | - | - | - | - | - |
| ***S. pneumoniae*** | 86% (147/171) | - | - | 88%  (37/42)* | - | - | - | - | - | - | - |
| **Beta-haemolytic *Streptococci*** | 100% (108/108) | - | - | - | - | - | - | - | - | - | - |
| ***S. bovis* group** | 100% (22/22) | - | - | - | - | - | - | - | - | - | - |
| ***E. faecalis*** | NA | - | 99.5% (225/226) | - | 100% (197/197) | 98.7% (230/233) | 97.8% (89/91) | 95.45% (168/176) | - | - | 54.6% (107/196) |
| ***E. faecium*** | NA | - | 6.6% (10/151) | - | 92.0% (127/138) | 67.7% (109/161) | NA | 98.5% (133/135) | - | - | 40% (52/130) |
| ***L. monocytogenes*** | 71.4% (10/14) | - | 100% (12/12) | - | - | - | - | - | - | - | - |

*5/42 strains CRO-intermediate, 1/42 CRO-resistant. PEN = Penicillin; FLU = Flucloxacillin; AMP = Ampicillin; CRO = Ceftriaxone; TEC = Teicoplanin; VAN = Vancomycin; DAP = Daptomycin; LZD = Linezolid; RIF = Rifampicin; CIP = Ciprofloxacin; GEN = Gentamicin.

**Supplementary table 3. Antimicrobial resistance in BSI isolates, overall and according to neutropenic status**

| **Causative pathogen and** |  | **Resistant isolates** |  | **p-value** |
| --- | --- | --- | --- | --- |
| **antimicrobial class** | **Total** | **Neutropenia** | **Non neutropenia** |  |
| ***E. coli*** |  |  |  |  |
| Ampicillin | 727/1288 (56.4%) | 455/769 (59.2%) | 272/519 (52.4%) | 0.016 |
| Amoxicillin-clavulanate | 289/1277 (22.6%) | 190/762 (24.9%) | 99/514 (19.2%) | 0.018 |
| 3GC (any) | 104/1285 (8.1%) | 68/767 (8.9%) | 36/518 (6.9%) | 0.22 |
| Ciprofloxacin | 136/1264 (10.8) | 94/758 (12.4%) | 42/506 (8.3%) | 0.021 |
| Piperacillin-tazobactam | 79/1005 (7.9%) | 57/622 (9.2%) | 22/383 (5.7%) | 0.050 |
| TMP-SXT | 557/1281 (43.5%) | 366/764 (47.9%) | 191/517 (36.9%) | <0.001 |
| ***Klebsiella* spp.** |  |  |  |  |
| Amoxicillin-clavulanate | 74/773 (9.6%) | 52/538 (9.7%) | 22/233 (9.4%) | 0.89 |
| 3GC (any) | 45/776 (5.8%) | 5.8% (31/539) | 14/237 (5.9%) | 0.93 |
| Ciprofloxacin | 34/767 (4.4%) | 24/537 (4.4%) | 10/230 (4.3%) | 0.94 |
| Piperacillin-tazobactam | 61/625 (9.8%) | 47/449 (10.5%) | 14/176 (8.0%) | 0.34 |
| TMP-SXT | 170/774 (22%) | 128/537 (23.8%) | 42/237 (17.7%) | 0.058 |
| **ESCPM** |  |  |  |  |
| 3GC R (any) | 178/694 (27.5%) | 112/434 (27.9%) | 66/260 (25.3%) | 0.74 |
| Ciprofloxacin | 31/640 (4.8%) | 20/393 (5.1%) | 11/247 (4.5%) | 0.72 |
| Piperacillin-tazobactam | 79/499 (15.8%) | 48/314 (15.3%) | 31/185 (16.8%) | 0.66 |
| TMP-SXT | 166/646 (25.7%) | 123/399 (30.8%) | 43/247 (17.4%) | <0.001 |
| Carbapenem | 8/641 (1.2%) | 5/399 (1.3%) | 3/242 (1.2%) | 0.99 |
| ***Pseudomonas* spp.** |  |  |  |  |
| Ciprofloxacin | 52/1195 (4.4%) | 37/819 (4.5%) | 15/376 (4%) | 0.68 |
| Ceftazidime | 82/1162 (7.1%) | 50/800 (6.3%) | 32/362 (8.8%) | 0.11 |
| Piperacillin-tazobactam | 81/919 (8.8%) | 56/653 (8.6%) | 25/266 (9.4) | 0.69 |
| Carbapenem | 46/1184 (3.9%) | 32/813 (3.9%) | 14/371 (3.8%) | 0.89 |
| **Non-Fermenting Gram-negatives**^a^ |  |  |  |  |
| Carbapenem | 77/1430 (5.4%) | 45/938 (6.5%) | 32/492 (5.5%) | 0.17 |
| ***S. maltophila*** |  |  |  |  |
| TMP-SXT | 11/204 (5.4%) | 10/125 (8.0%) | 1/79 (1.3%) | 0.038 |
| ***S. aureus*** |  |  |  |  |
| Flucloxacillin (MRSA) | 153/824 (18.6%) | 50/273 (18.3%) | 103/551 (18.7%) | 0.9 |
| **CoNS** |  |  |  |  |
| Flucloxacillin | 686/798 (86.0%) | 233/280 (83.2%) | 453/518 (87.5%) | 0.1 |
| ***Streptococcus* spp.^b^** |  |  |  |  |
| Penicillin^c^ | 79/496 (15.9%) | 51/236 (21.6%) | 28/260 (10.8%) | <0.001 |
| ***Enterococcus* spp.^d^** |  |  |  |  |
| Vancomycin | 55/394 (14.0%) | 46/250 (18.4%) | 9/144 (6.3%) | <0.001 |

BSI = Bloodstream infection; 3GC = 3^rd^ generation cephalosporin; TMP-SXT = trimethoprim-sulphametozaxole; ESCPM = *Enterobacter spp., Serratia marcescens, Citrobacter freundii, Providencia* spp. and *Morganella morganii*. MRSA = methicillin-resistant *S. aureus;* CoNS =*Coagulase Negative Staphylococci.* ^a^Including *Pseudomonas* spp., *Acinetobacter* spp., *Achromobacter* spp., *Burkholderia* spp. *Sphingomonas* spp. *Chryseobacterium* spp.; ^b^Including all *Streptococcus* spp. ^c^Including intermediate strains; ^d^Including *E. faecalis* and *E. faecium.*

**Supplementary figure 1: Kaplan-Meier curves for 30-day case-fatality according to neutropenic status (log-rank testp<0.001)**


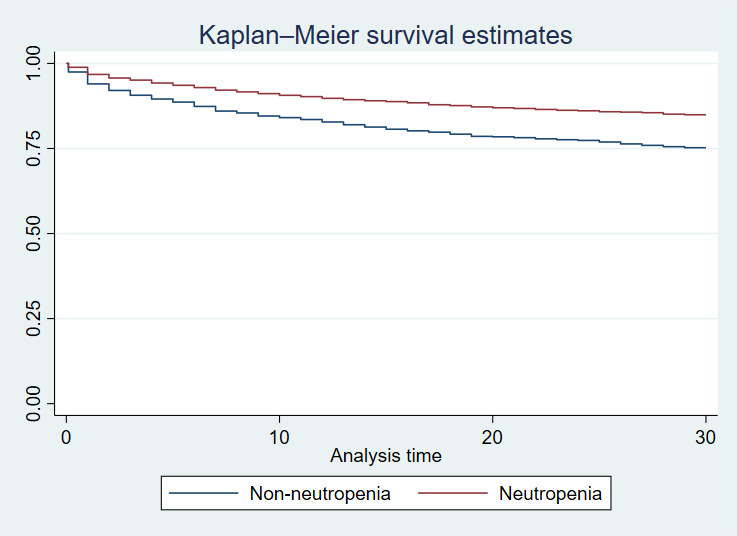

Supplement: Supplementary file 1 — (DOCX 72 KB) [file 10238_2023_1206_MOESM1_ESM.docx]
